# B cell receptor–based hybridoma enrichment enables discovery of antibodies against intracellular tumor antigens

**DOI:** 10.3389/fimmu.2026.1867422

**Published:** 2026-07-30

**Authors:** Iris Dotan, Yuval Raviv, Débora R. Bublik, Jule A. Kortendieck, Shrey Jain, Yifat Glucksam Galnoy, Nechama I. Smorodinsky, Adi Barzel

**Affiliations:** 1 School of Neurobiology, Biochemistry and Biophysics, The George S. Wise Faculty of Life Sciences, Tel Aviv University, Tel Aviv, Israel; 2 The Shmunis School of Biomedicine and Cancer Research, The George S. Wise Faculty of Life Sciences, Tel Aviv University, Tel Aviv, Israel; 3 The Varda and Boaz Dotan Center for Advanced Therapies at the Tel Aviv Sourasky Medical Center, Tel Aviv, Israel

**Keywords:** antibody discovery, B cell receptor (BCR), B cell responses, hybridoma technology, immunotherapy, intracellular tumor antigens, monoclonal antibodies, tumor immunology

## Abstract

Tumor-associated B cell responses are frequently observed in tumors and their draining lymph nodes, yet their antigen specificities and functional potential remain incompletely defined. Historically, intracellular antigens have been largely disregarded in tumor antigen mining and discovery efforts. In particular, the extent to which intracellular tumor antigens can be effectively targeted by antibodies and the cellular immune system has remained unclear, poorly characterized, and therapeutically underexploited. We developed a high-throughput platform combining hybridoma generation with unbiased antigen-based flow cytometric enrichment to isolate tumor-reactive monoclonal antibodies. Using a murine 4T1 triple-negative breast cancer model engineered to express the intracellular protein ZsGreen1 (ZsG), we characterized B cell responses and selected antigen-specific hybridomas via fluorescence-activated cell sorting. Antibody specificity was validated using biochemical and cellular assays, and *in vivo* activity was evaluated following systemic administration. Translational applicability was assessed using hybridomas generated from human tonsillar and peripheral blood B cells. Tumor growth elicited a class-switched, antigen-specific B cell response against the intracellular ZsG antigen. Hybridomas retained a dual phenotype, expressing both surface B cell receptors and secreted immunoglobulins, enabling efficient antigen-driven selection. Isolated monoclonal antibodies recognized native intracellular epitopes that were not detectable using conventional screening approaches. Notably, systemic administration of an anti-ZsG antibody was associated with reduced tumor growth in early established tumors *in vivo*, supporting functional antibody activity under these conditions. Furthermore, biotinylated tumor lysates enabled unbiased identification of additional tumor-reactive antibodies, and the platform was successfully extended to human B cells. These findings support the accessibility of intracellular tumor antigens to antibody recognition and establish a framework for discovering antibodies targeting this antigen class. The platform described here provides a scalable approach for isolating tumor-reactive antibodies and expands the landscape of potential targets for cancer immunology and immunotherapy.

## Introduction

Tumor-infiltrating B cells (TIL-Bs) arise at multiple stages of tumorigenesis and play diverse roles in shaping the tumor microenvironment (TME) (1–6). Identified across approximately 60% of breast cancers and 77% of primary melanomas, these cells often organize into tertiary lymphoid structures (TLS), where mature B cells and differentiated plasma cells correlate with improved survival and reduced relapse rates in multiple solid tumors (5, 7–14). Despite these positive correlations, the specific antigen targets and the full therapeutic potential of the host B cell response remain underexplored.

Traditionally, the development of monoclonal antibody (mAb) therapy has prioritized membrane-associated or extracellular targets to facilitate direct effector functions, such as antibody-dependent cellular cytotoxicity (ADCC) or complement-dependent cytotoxicity (CDC) (15–20). However, a substantial portion of the naturally occurring humoral response in cancer patients, is directed against the intracellular proteome i.e. proteins localized to the cytoplasm or nucleus (2, 21–24). For example, clinical evidence indicates that the presence of serum autoantibodies against NY-ESO-1 in melanoma and non-small-cell lung carcinoma (NSCLC), together with expanded CD8^+^ responses, is associated with a favorable response to anti-CTLA-4 checkpoint immunotherapy treatment (25). Similarly, antibodies targeting the E6 and E7 oncoproteins of Human Papillomavirus (HPV) in head and neck cancers have been linked to improved clinical outcomes, suggesting these elicited antibodies are not merely markers of cell death but rather active participants in tumor control (26–28).

While it is often assumed that sequestered antigens are inaccessible to systemic antibodies, the correlation between antibodies elicited against these antigens and positive prognosis suggests an underlying mechanism of action. It is hypothesized, that intracellular antigens released into the TME through necrosis, apoptosis, or active secretion in exosomes, can form immune complexes with cognate antibodies (3, 29–31). These complexes facilitate Fc-receptor-mediated internalization by professional antigen-presenting cells (APCs), such as dendritic cells (31). This process enhances the priming of CD4^+^ T cells via MHC Class II and the cross-presentation of antigens on MHC Class I to license CD8^+^ cytotoxic T cell responses, a phenomenon known as the “vaccinal effect” of antibodies (31, 32).

A major bottleneck in exploiting this biology is the lack of high-throughput methodologies to isolate antigen-specific B cell repertoires of the host. Hybridoma technology remains a widely used B cell immortalization method, providing robust antibody-secreting capacity (33–39). Yet, traditional hybridoma screening relies mainly on antibody secretion and labor-intensive, stochastic methods, like ELISA. These traditional methods are poorly suited for mining complex, low-frequency tumor-reactive repertoires, especially those targeting native and conformational epitopes. More recent approaches based on flow cytometry sorting and membranal BCRs expressed on the cell surfaces of hybridomas, enable the isolation and cloning of mAbs in a far more rapid and efficient protocol, compared to past approaches (40, 41).

In this study, we describe an unbiased high-throughput pipeline that transforms the hybridoma from a simple “antibody factory” into a selectable sensor. We demonstrate that hybridomas derived from early B cell responders in the tumor-draining lymph nodes (TDLNs) of mice bearing 4T1 triple-negative breast cancer (TNBC) retain a dual phenotype: the concomitant expression of membrane-bound B cell receptors (BCR) and secreted antibodies. This unique feature enables rapid, antigen-specific enrichment via flow cytometry. By applying this pipeline to a model intracellular antigen ZsG (42), we reveal a functional humoral response against tumor-specific sequestered targets that mimics the prognostic responses seen in human patients, thus establishing a generalizable platform for the discovery of therapeutically-relevant tumor-directed antibodies.

## Results

### Tumor growth induces a class-switched B cell response in tumor-draining lymph nodes

**Figure 1:**
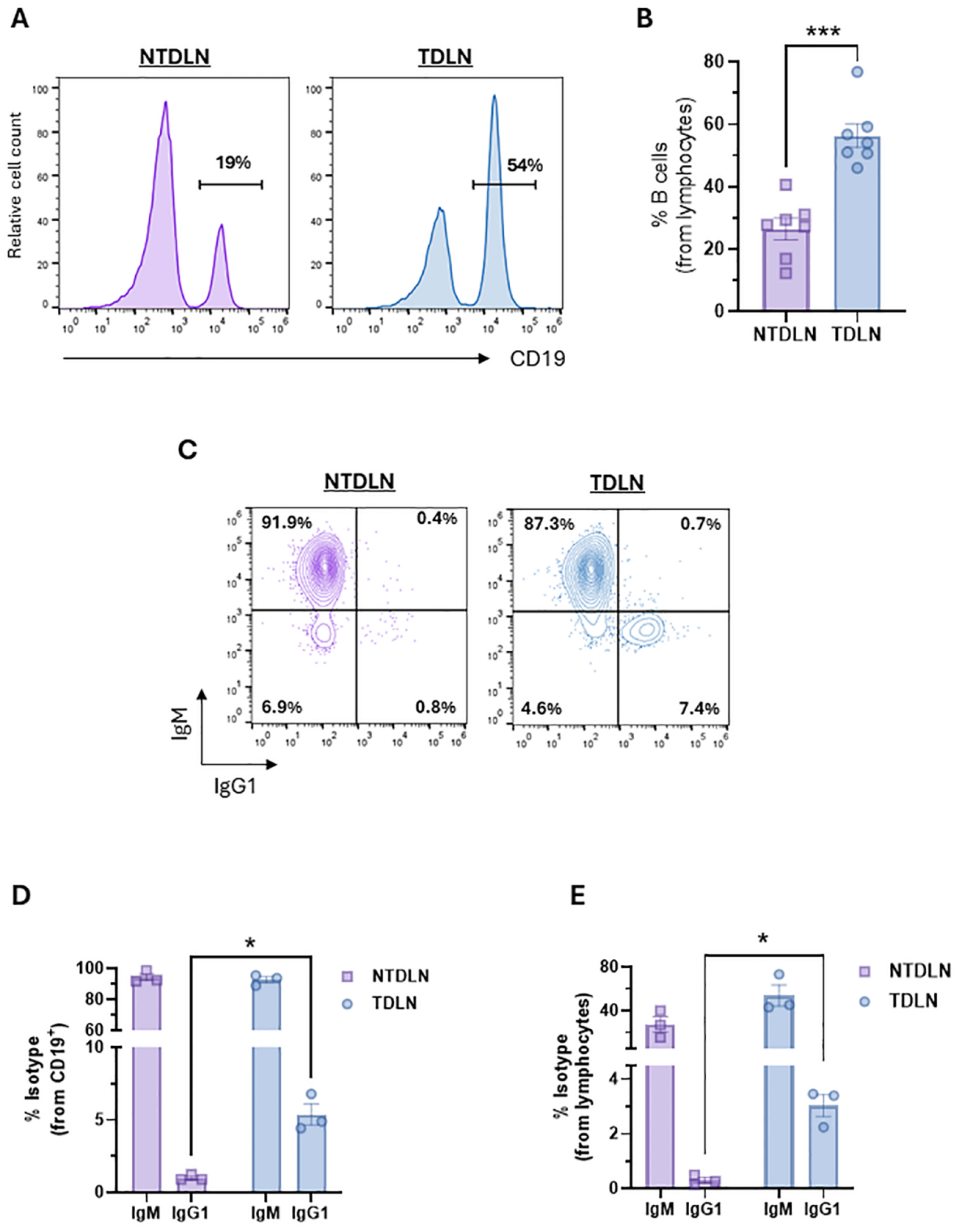
Characterization of tumor draining lymph nodes (TDLN) and non-tumor draining lymph nodes (NTDLN) in mice bearing 4T1 tumor. **(A)** Representative flow cytometry analysis of CD19^+^ B cell abundance in TDLN of a mouse orthotopically engrafted with 4T1 tumor cells (right panel), compared to the distal cervical NTDLN of the same mouse (left panel). **(B)** Percentage of B cells out of total lymphocytes in TDLN vs distal cervical NTDLN, in mice orthotopically engrafted with 4T1 tumor cells. n=7. Error bars represent standard error. ***p<0.001, two-tailed t-test. **(C)** Representative flow cytometry analysis of IgM and IgG1 expression among CD19^+^ B cells in the TDLN of a mouse orthotopically engrafted with 4T1 tumor cells (right panel), compared to the distal cervical NTDLN of the same mouse (left panel). **(D)** Proportion of IgM^+^ and IgG1^+^ out of CD19^+^ B cells in TDLNs vs. NTDLNs on day 14–18 post tumor engraftment. n=3. Error bars represent standard error. *p<0.05, two-tailed t-test. **(E)** Percentage of IgM^+^ and IgG1^+^ out of total lymphocytes in TDLNs vs. NTDLNs of day 14–18 post tumor engraftment. n=3. Error bars represent standard error. *p<0.05, two-tailed t-test.

To investigate tumor-associated B cell responses *in vivo*, female BALB/c mice were orthotopically implanted with 4T1 TNBC cells. Fourteen to twenty days after tumor engraftment, TDLNs exhibited a marked enrichment of CD19^+^ B cells compared with distal lymph nodes, indicating an active humoral response to the developing tumor (**Figures 1A, B**; **Supplementary Figure 1**).

While IgM^+^ B cells constituted the majority of the B cell compartment in both lymph node types (>90%), a pronounced enrichment of class-switched B cells was observed specifically in TDLNs (**Figures 1C, D**). IgG1^+^ B cells represented approximately 5.4% of B cells in TDLNs, compared with ~1% in distal lymph nodes, corresponding to a greater than fivefold relative increase. Because TDLNs also contained approximately twice the total number of CD19^+^ B cells, this translates into an estimated tenfold increase in the absolute number of IgG1^+^ B cells in TDLNs, relative to distal nodes (**Figure 1E**).

### Engineering a traceable intracellular tumor antigen model

**Figure 2:**
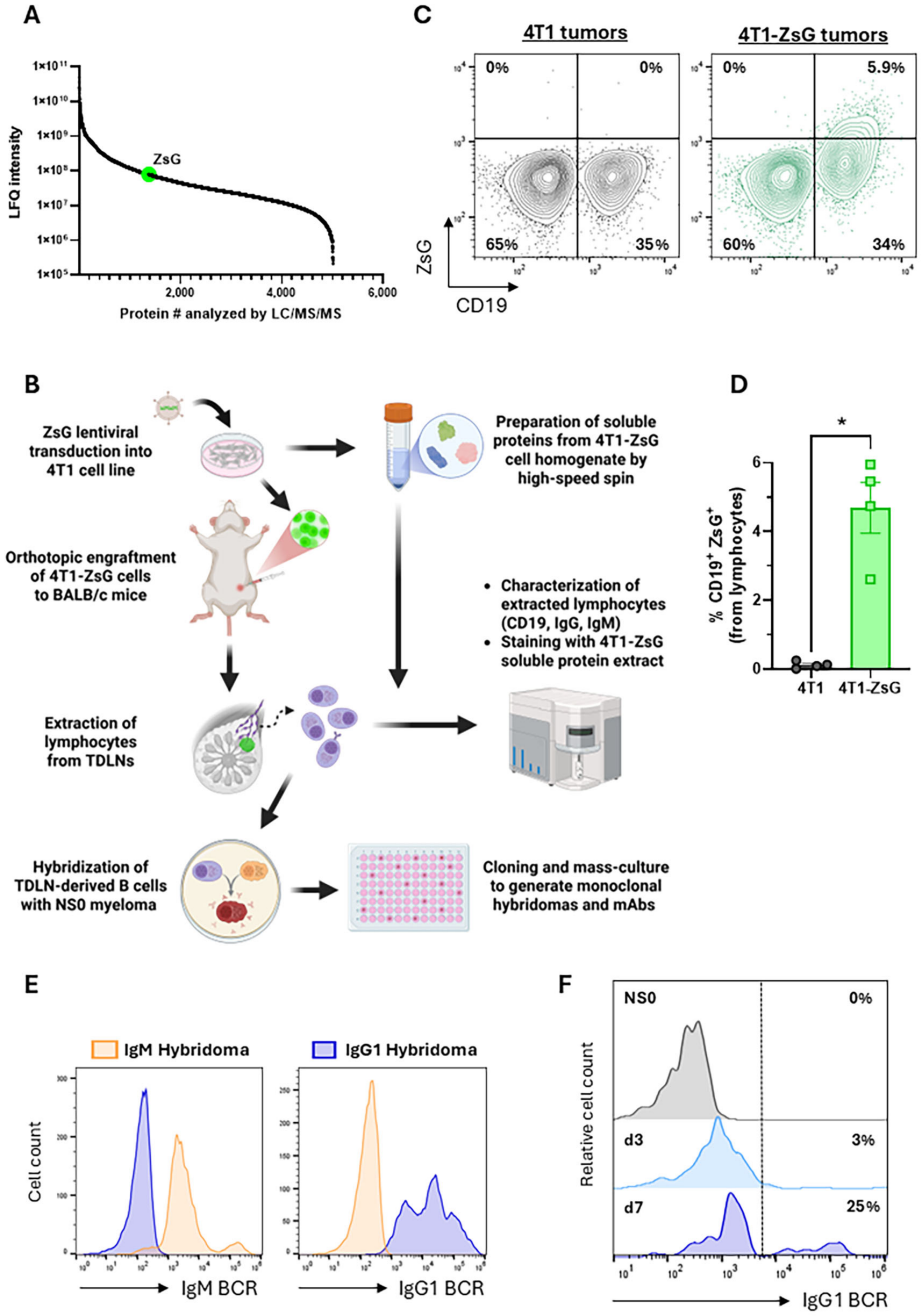
Characterization of 4T1-ZsG tumors and their TDLNs. **(A)** Proteomic analysis of the abundance of ZsG in 4T1-ZsG cells, relative to other detected cellular proteins, as measured by intensities of 2D mass spectrometry (LC-MS/MS). **(B)** Experimental scheme depicting the generation of cloned hybridomas from TDLN-derived lymphocytes of mice engrafted orthotopically with 4T1-ZsG cells. **(C)** Representative flow cytometry analysis of binding of soluble extract prepared from 4T1-ZsG tumor cells by lymphocytes in the TDLN of mice orthotopically engrafted with 4T1-ZsG cells. **(D)** Proportion of CD19^+^ ZsG^+^ B cells in the TDLN of 4T1 vs. 4T1-ZsG, on days 14–18 after tumor engraftment. n=4. Error bars represent standard error. *p<0.05, two-tailed t-test. **(E)** Representative flow cytometry analysis measuring the expression of IgM BCR on hybridoma (orange) versus an IgG1 BCR (blue), in two monoclonal hybridomas generated from TDLN lymphocytes. Each panel represents a prior mixture of the 2 hybridomas, stained either with mouse IgM antibody (left panel) or mouse IgG1 antibody (right panel). **(F)** Representative flow cytometry analysis tracking the temporal appearance of BCRs (IgG1) on TDLN-derived hybridomas at the indicated days (d) post hybridization. Mouse myeloma cell line (NS0) served as control.

To study B cell responses against an intracellular tumor antigen, a fluorescent model antigen was introduced into the 4T1 tumor system. The ZsG (42) gene was stably expressed in 4T1 cells by lentiviral transduction, generating the 4T1-ZsG cell line. Flow cytometry confirmed ZsG expression in 97% of transduced cells (**Supplementary Figure 2**). Further enrichment of ZsG-expressing cells to 100% was attained by FACS. To assess the relative abundance of ZsG within tumor cells, detergent-solubilized whole-cell lysates from 4T1-ZsG cells were analyzed by liquid chromatography-tandem mass spectrometry (LC-MS/MS). Proteomic analysis demonstrated that ZsG is expressed in 4T1-ZsG cells at a moderate cellular abundance and is not among the most abundant endogenous proteins. Out of 5,202 proteins detected, ZsG ranked 1,376^th^ in relative abundance (**Figure 2A**). Thus, this proteomic analysis has established ZsG as a traceable, yet non-dominant intracellular tumor-specific antigen, absent from parental 4T1 cells.

### Intracellular tumor antigens elicit antigen-specific B cell responses in tumor-draining lymph nodes

Female BALB/c mice were implanted orthotopically with 4T1-ZsG cells and lymphocytes were isolated from their TDLNs 14–20 days later (**Figure 2B**). Consistent with an active germinal center response, the majority of B cells within TDLN germinal centers of 4T1-ZsG tumor expressed IgG1 (**Supplementary Figure 3**), implying ongoing class switching and affinity maturation against tumor-associated antigens.

To probe antigen specificity, lymphocytes derived from TDLNs of tumor-bearing mice (4T1 tumors and separately 4T1-ZsG tumors) were stained with a protein/antigen extract derived from a 4T1-ZsG fractionated cell homogenate (S_105_-4T1-ZsG), representing the soluble, high-speed spin supernatant, intracellular protein/antigen pool. Being a soluble protein, the ZsG fractionated alongside all the other soluble proteins of the 4T1-ZsG cell line and resided in the same collected high-speed-spin supernatant fraction (S_105_).

Flow cytometric analyses of the ZsG-stained TDLN lymphocytes revealed that a subset of CD19^+^ B cells in the TDLNs of 4T1-ZsG-tumor-bearing mice bound the ZsG fluorescent protein contained in the soluble extract of 4T1-ZsG cells, indicating the presence of ZsG-specific functional B cell receptors (BCRs) on those B cells (**Figures 2C, D**). In contrast, no comparable binding was detected in TDLNs from mice bearing parental 4T1 tumors, demonstrating antigen specificity. Thus, already in the TDLNs of 4T1-ZsG tumors, binding of the ZsG antigen proved sensitive enough to detect lymphocytes reacting against ZsG.

### Hybridomas derived from tumor-reactive B cells retain functional B cell receptors

Hybridomas were generated from TDLN-derived B cells using the NS0 myeloma fusion partner (**Figure 2B**). Resultant hybridomas exhibited a dual phenotype, characterized by concomitant single-isotype expression of both a membrane-bound BCR and a secreted immunoglobulin. BCR expression on a generated hybridoma pool increased with time after hybridization and was stably expressed on expanded clones (**Figures 2E, F**). The dual feature enabled direct interrogation of antigen specificity at the cellular level.

**Figure 3:**
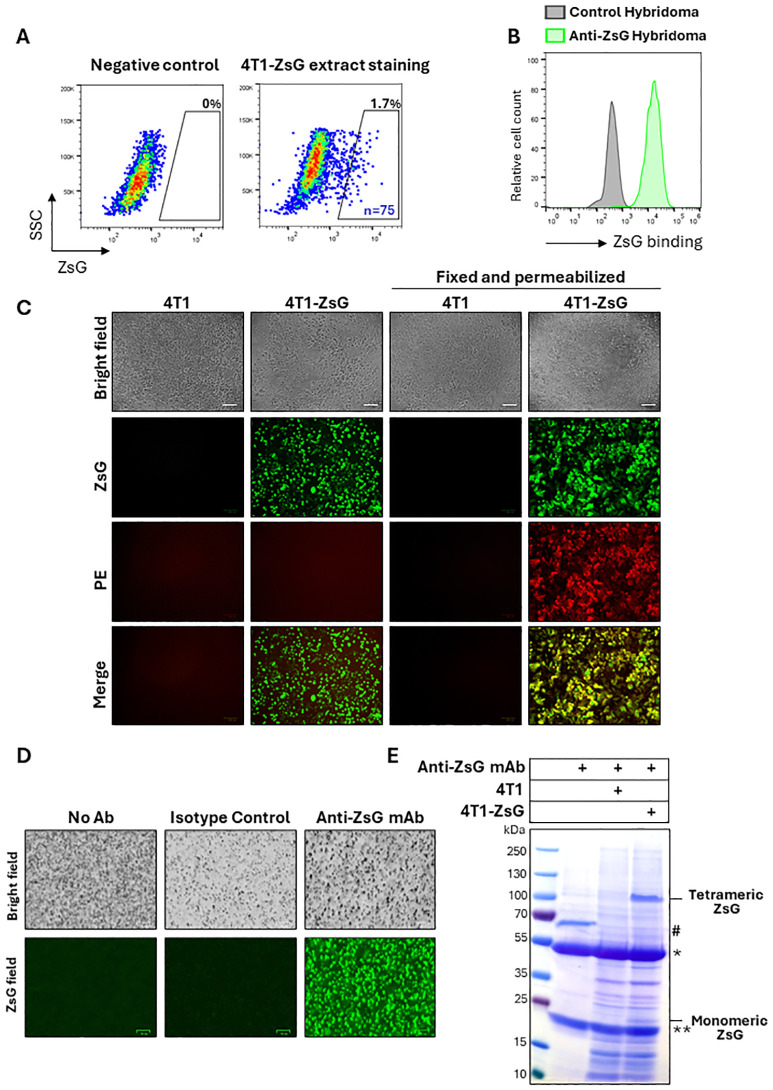
Sorting and characterization of anti-ZsG hybridomas and immunoprecipitation of the ZsG protein. **(A)** Representative flow cytometry sorting of hybridomas that best bind the ZsG in S_105_-4T1-ZsG, gated according to a control hybridoma (negative control). **(B)** Representative flow cytometry confirming stable and uniform ZsG fluorescence derived from 4T1-ZsG protein extract (S_105_-4T1-ZsG) binding to a sorted and cloned anti-ZsG hybridoma. **(C)** Representative fluorescent microscopy images of untreated, or fixed and permeabilized 4T1 and 4T1-ZsG cells, stained by protein A purified antibody from a cloned anti-ZsG IgG1 hybridoma, followed by anti-mouse IgG1-PE secondary antibody staining. Intrinsic ZsG fluorescence is shown in green and anti-ZsG immunostaining in red (PE). Colocalization of the ZsG and PE signals is shown in the merged images on the bottom row, where overlapping fluorescence appears yellow. Scale bar: 100 µm. **(D)** Representative fluorescent microscopy of Protein L beads loaded either with a monoclonal antibody against ZsG (anti-ZsG mAb), an isotype control antibody (IgG1), or no antibody (No Ab), and immunoprecipitated after incubation with S_105_-4T1-ZsG soluble protein/antigen extract. Scale bar: 25 µm. **(E)** SDS-PAGE of immunoprecipitation products with Protein L beads loaded with a mAb against ZsG and further incubated with either S_105_-4T1-ZsG soluble protein extract, S_105_-4T1 soluble protein extract, or no extract, stained with Coomassie blue. The differential annotated bands, conforming to the size of the ZsG protein in its monomeric and its tetrameric form, were excised and sent to 2D mass spectrometry (LC-MS/MS). Bands conforming to the size of BSA (#), mouse IgG1 heavy (*) and light chains (**), are marked.

ZsG-binding hybridomas derived from TDLNs of mice bearing 4T1-ZsG tumors were enriched using fluorescence-activated cell sorting (FACS) based on binding of soluble ZsG-containing extracts to the BCR (**Figure 3A**). To increase the frequency and affinity of extract-binding-hybridomas, a prior enrichment step was performed, with IgG1 MACS^®^ columns, using a pool of newly hybridized cells. Single cells corresponding to high binders of ZsG, were then sorted into individual wells and expanded to generate independent monoclonal hybridoma lines.

Monoclonal hybridomas isolated through this strategy bound ZsG directly via their BCRs (**Figure 3B**) and secreted antibodies. When these antibodies were expanded in bioreactors and purified by Protein A affinity chromatography, they exhibited robust and specific staining of 4T1-ZsG cells (but not parental 4T1 cells). This staining was observed only after the cells were fixed and permeabilized, consistent with the strict intracellular compartmentalization of the ZsG model antigen (**Figure 3C**). Notably, these antibodies failed to produce signal in multiple ELISA formats, including covalent antigen-capturing solid supports, suggesting recognition of conformational epitopes which are usually disrupted by standard coating procedures. These findings indicate that conventional ELISA-based screening pipelines may systematically overlook antibodies directed against native intracellular tumor antigens.

### Identification and molecular target validation of intracellular antigen-specific monoclonal antibodies

To confirm antigen specificity, secreted antibodies were captured using Protein L beads and incubated with ZsG-containing cell extracts. Beads loaded with anti-ZsG monoclonal antibodies exhibited robust fluorescence upon incubation with 4T1-ZsG extracts, whereas beads loaded with isotype control antibodies or no antibody did not (**Figure 3D**). Thus, bulk binding of the ZsG antigen by the immobilized antibody was accompanied by retention of ZsG fluorescence, making it a convenient method to identify and track anti-ZsG antibodies.

Immunoprecipitation followed by SDS-PAGE revealed protein bands corresponding to the expected molecular weights of monomeric form of ZsG, as well as the known predominant, denaturation-resistant tetrameric form (**Figure 3E**). LC-MS/MS analysis of the putative monomer and tetramer excised bands confirmed ZsGreen as the dominant cellular protein in these bands (**Supplementary Appendix A**). Sequencing of two representative immunoglobulin heavy variable regions of anti-ZsG monoclonal antibodies used in such immunoprecipitations revealed somatic mutations consistent with *in vivo* affinity maturation of the source lymphocytes used for hybridization (**Supplementary Figure 4**).

### A BCR-based hybridoma enrichment strategy enables unbiased discovery of tumor-reactive antibodies

**Figure 4:**
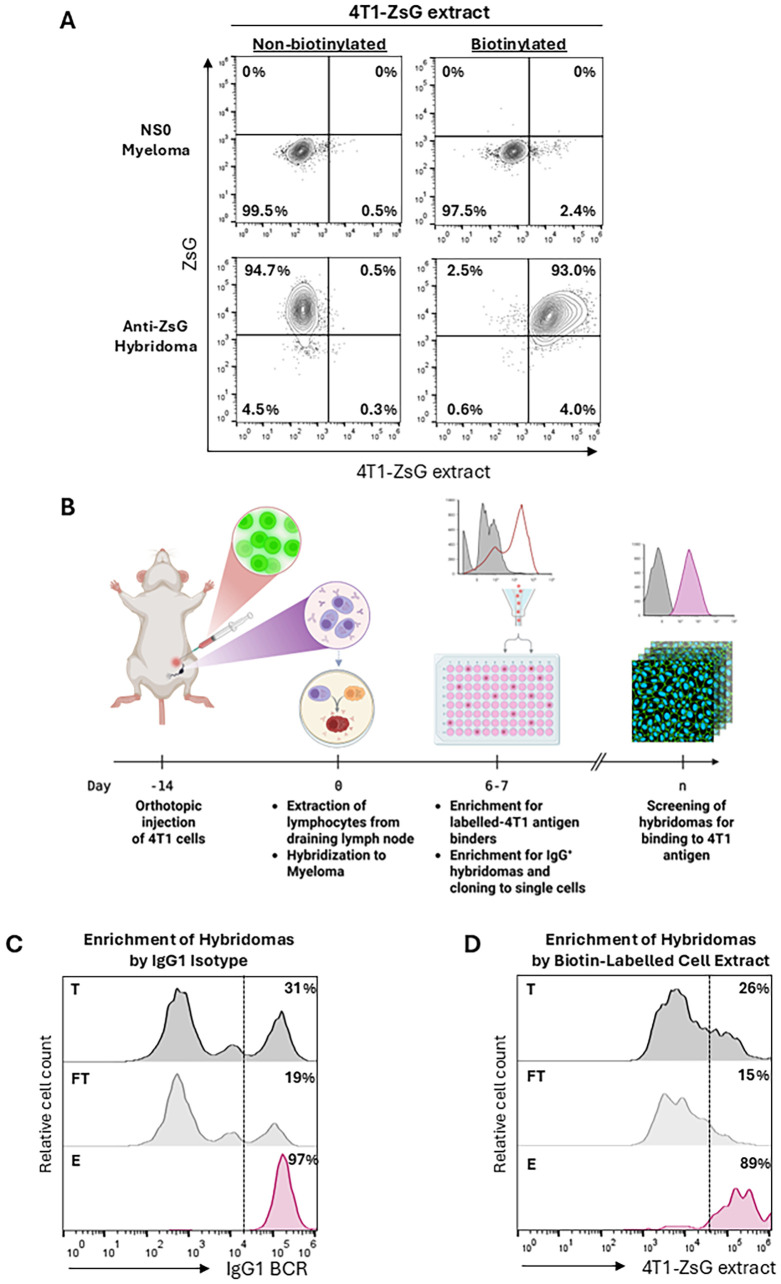
Characterization of the binding properties of hybridomas to biotinylated 4T1 and 4T1-ZsG cell extracts. **(A)** Flow cytometry analysis of anti-ZsG hybridoma cells (bottom panels) compared to the NS0 myeloma (upper panels), following incubation with biotinylated 4T1-ZsG soluble protein/antigen extract (right panels) or non-biotinylated extract (left panels). Bound biotinylated extract was detected using Streptavidin-PE. **(B)** Optimized pipeline scheme for enriching hybridomas based on their membranal expression of a BCR. **(C)** Representative flow cytometry analysis of the total (T), flowthrough (FT) and eluted (E) fractions following MACS^®^ column enrichment of hybridomas that express IgG1 BCRs. **(D)** Representative flow cytometry analysis of the total (T), flowthrough (FT) and eluted (E) fractions of antigen-based MACS^®^ column enrichment of hybridomas using biotinylated soluble 4T1 cell extract.

To generalize this approach beyond defined model antigens, soluble tumor cell culture extracts were unbiasedly labeled, using maleimide-biotin chemistry. Labeled extracts were then used to probe hybridoma pools. As proof of concept for this strategy, an isolated anti-ZsG monoclonal hybridoma was shown to be efficiently stained by biotin-labeled 4T1-ZsG extracts, exhibiting a concomitant high signal in both the ZsG fluorescent channel and the Streptavidin-Biotin channel. NS0 myeloma cells, lacking BCR expression, showed no binding (**Figure 4A**). For unknown labeled antigens (**Figure 4B**), MACS^®^-based enrichment using both anti-IgG isotype nanobeads (**Figure 4C**) and streptavidin nanobeads (**Figure 4D**) was implemented to increase the frequency of biotinylated extract-binding hybridomas.

Following single-cell cloning, antibodies secreted from 8 of 46 (17%) monoclonal hybridomas isolated using this strategy exhibited distinct intracellular staining patterns of 4T1 tumor cells. These hybridoma clones spanned multiple isotypes, including IgG1, IgG2ab, and IgM (**Supplementary Table 1**). Overall, this approach substantially exceeded rates and yields typically achieved using conventional immunization and screening pipelines.

### Antibodies targeting an intracellular tumor antigen inhibit tumor growth *in vivo*

**Figure 5:**
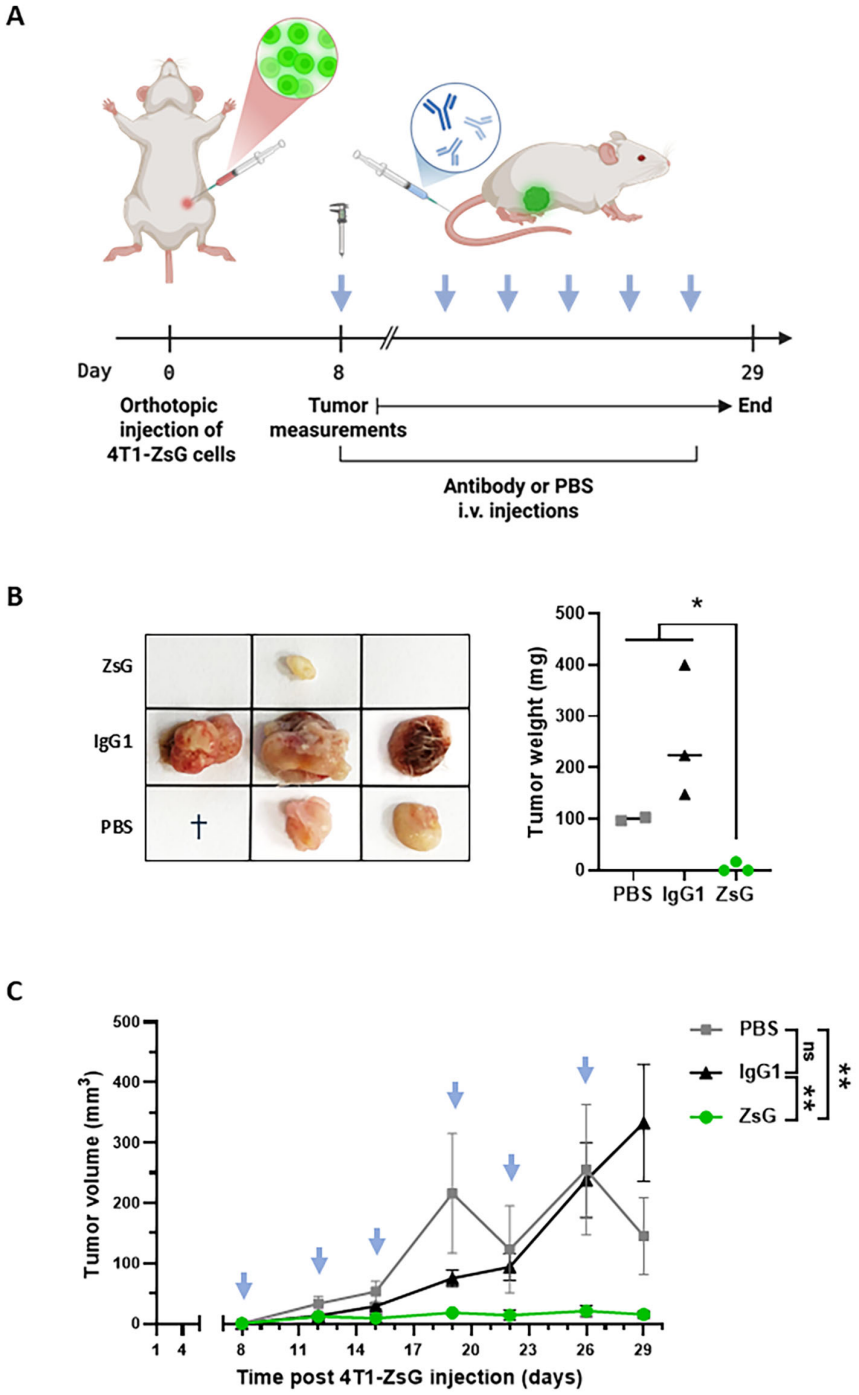
Effect of anti-ZsG antibody injections on mice bearing 4T1-ZsG tumors. **(A)** Experimental scheme for anti-ZsG antibody, IgG1 antibody, or PBS i.v. injections twice weekly (light blue arrows), starting on day 8 post 4T1-ZsG tumor engraftment, when tumors were palpable, in female BALB/c mice. n=3 mice per group. **(B)** Primary tumors from the *in vivo* assay described in A, as extracted (left) and weighed (right) at endpoint. Each dot represents an individual mouse and bars indicate the median. Statistical significance between ZsG and pooled controls (PBS and IgG1) was determined using a Mann-Whitney U test. *p < 0.05. ^†^ denotes a PBS-treated animal that died before the study’s endpoint. Empty panels indicate mice in which there was no grossly detectable tumor at endpoint, neither by palpation nor by caliper measurement. **(C)** Primary tumor size growth curves, as measured using a caliper (tumor volume is expressed in mm^3^ calculated using the formula 0.5*Length*Width^2^). Light blue arrows represent injection time points. Values are mean of measured tumor sizes in each group. n=3. Error bars represent standard error. **p<0.01, ns, not significant, two-way ANOVA.

To assess the therapeutic relevance of antibodies targeting intracellular tumor antigens, two monoclonal antibodies were produced at scale: an IgG1 antibody specific for ZsG and an IgG1 control antibody recognizing an unrelated intracellular antigen that is not specific to tumor cells. These two monoclonal antibodies were produced by hybridomas that were isolated by the above-described pipeline. Specificity of the anti-ZsG antibody was demonstrated as described, by selective staining of fixed/permeabilized 4T1-ZsG cells but not parental 4T1 cells, by binding to ZsG-containing extracts, and by IP followed by LC-MS/MS confirmation of ZsG. Treatment with the monoclonal antibodies was initiated when 4T1-ZsG tumors became palpable. Mice received biweekly intravenous injections of anti-ZsG antibody, the IgG1 control antibody, or PBS, according to the indicated dosing schedule (**Figure 5A**).

Anti-ZsG antibody treatment resulted in marked inhibition of tumor mass. At the experimental endpoint, two of three anti-ZsG-treated mice exhibited complete tumor elimination, while the third displayed a substantially reduced tumor burden. In contrast, PBS-treated mice developed large tumors, and one animal succumbed prior to study completion. Notably, mice treated with the control IgG1 antibody exhibited accelerated tumor growth (**Figure 5B**). Twice-weekly tracking of tumor size, in mice across all groups, showed that the endpoint was reflective of a monotonic effect. Thus, the anti-ZsG antibody exerted its inhibition from the earliest point of injection, whereas IgG1 and PBS treatments resulted in continuous growth of 4T1-ZsG tumors (**Figure 5C**). All mice that were alive at the endpoint were euthanized according to institutional ethical guidelines. These results demonstrate that systemic administration of antibodies targeting an intracellular tumor-specific antigen can mediate potent anti-tumor effects *in vivo.* A sample of tumors excised at endpoint were subjected to Collagenase and DNaseI treatment. Tumor-dissociated cells showed persistent expression of ZsG, as evaluated by flow cytometry (data not shown).

### Translation of BCR-based hybridoma enrichment to human B cells

**Figure 6:**
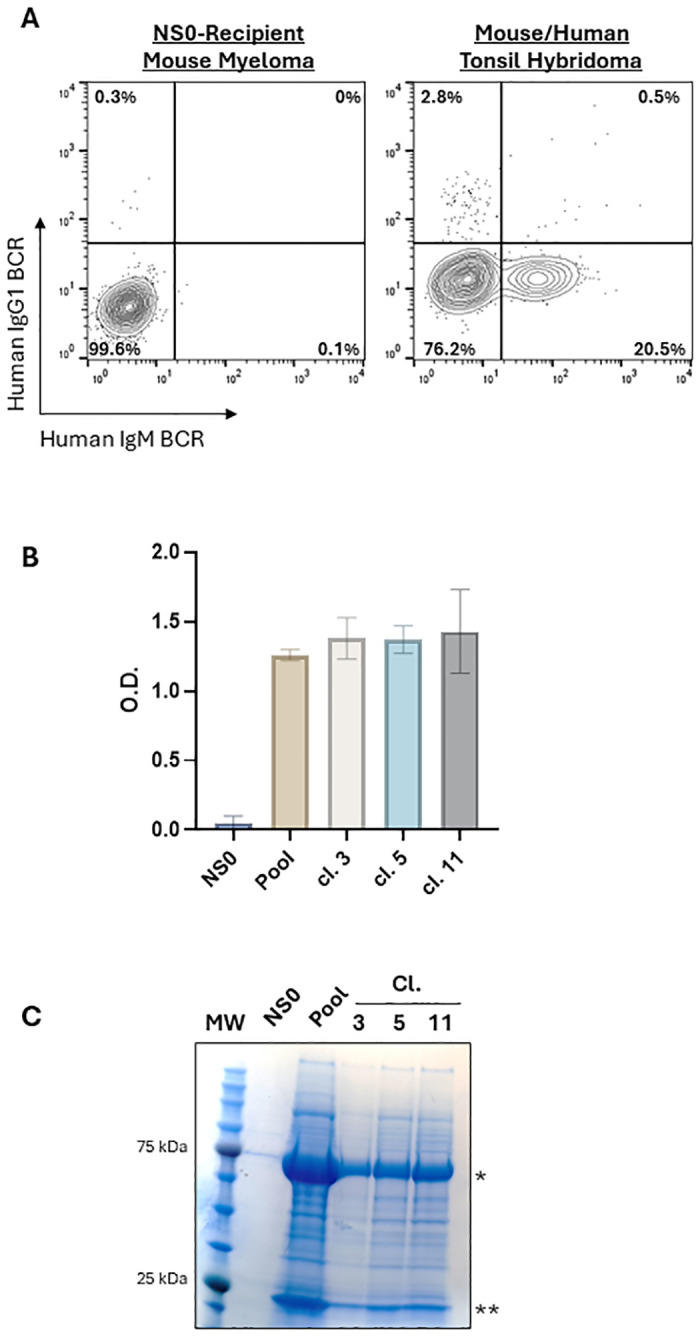
Characterization of human hybridomas generated by heterologous hybridization of human tonsil-derived cells with mouse NS0 myeloma. **(A)** Representative flow cytometry analysis of IgM and IgG1 expression in heterologous hybridization products of human tonsil-derived cells with NS0 mouse myelomas cell line, on day 12 after hybridization. **(B)** Secretion of antibodies from tonsil-derived single-cell human hybridomas (Cl. 3, 5 and 11), from a pool of them (Pool) or from the mouse NS0 myeloma fusion partner cells (NS0), analyzed by ELISA. Error bars represent standard deviation of triplicates. **(C)** Coomassie blue staining of SDS-PAGE loaded with denatured and SH-reduced samples of antibodies concentrated from serum-free supernatants of human monoclonal hybridomas. Heavy (*) and light chains (**) are marked. MW: molecular weight marker.

To explore translational feasibility, heterologous hybridomas were generated by fusing murine NS0 myeloma cells with human tonsillar cells, or human peripheral blood mononuclear cells (PBMCs) (43, 44). This was performed by emulating the hybridization protocol described above for the NS0 myeloma recipient partner and the human sourced cells. Following hybridization, flow cytometric analyses confirmed expression of human IgM and IgG BCRs on a substantial fraction of hybridomas derived from both sources (**Figure 6A**; **Supplementary Figure 5**). Single-cell cloning of tonsil-derived hybridomas also demonstrated robust secretion of fully assembled human monoclonal antibodies, as confirmed by ELISA and SDS-PAGE (**Figures 6B, C**). Thus, like murine TDLN cells, both human tonsillar cells and human PBMCs lend themselves to heterologous hybridization. These results establish that BCR-based enrichment strategies can be applied to human B cells using heterologous fusion partners, supporting the feasibility of isolating human monoclonal antibodies against intracellular tumor antigens from tumor-associated lymphoid tissues.

## Discussion

We present a pipeline for the unbiased, rapid isolation of tumor-targeting antibodies from the tumor host, along with a potential novel anti-tumor monoclonal antibody (mAb) therapy that leverages the host’s endogenous antibody response against intracellular tumor antigens. While most research has focused on membrane-bound or exosomal antigens, we specifically targeted subsets of antibodies directed against soluble intracellular antigens, a population previously shown to contribute to the host anti-tumor B cell immune response (2, 3, 21, 22, 45).

Existing literature has largely focused on isolating B cells specific for known, pre-defined antigens using labeled targets (40, 41, 46–53). Hybridomas have mostly been used in low-throughput, labor-intensive methods to isolate B cells and antibodies against tumor antigens such as Her2 and EGFR (33, 54–58). In contrast, we propose a novel high-throughput approach. By using the robust expression of B cell receptors on hybridomas as an antibody display platform, our workflow rapidly identifies tumor-targeting antibodies against unknown intracellular tumor antigens, without any prior target characterization.

We exploited the concomitant dual expression of BCR and secreted antibodies to screen hybridomas for their target antigens. This dual phenotype alludes to the parallel usage of both polyadenylation signals of the immunoglobulin transcript, expressed by the hybridoma cells. Possibly, this duality results from a combination of traits bequeathed to the hybridomas, by both the NS0 cells used as the hybridization fusion partner and activated BCR-bearing B cells from the TDLN. Specifically, BCR expression on hybridomas allows the bypass of traditional screening methodologies and can be applied to uncloned cell pools post-hybridization.

While conventional approaches seek extracellular tumor targets, we sought to model a soluble intracellular tumor target. For that purpose, we have expressed ZsG fluorescent protein in the murine 4T1 TNBC model cell line. ZsG is a codon optimized version of ZsGreen, which originates from a remote taxon (*Zoanthus* sp., reef coral) (42, 59). It most likely exhibits intrinsic immunogenicity and “foreign” antigen epitopes. We acknowledge that ZsG represents a model intracellular antigen and may exhibit immunogenic features distinct from endogenous tumor-associated antigens. Its use in this study was intended to provide a traceable system enabling rigorous validation of antigen specificity and functional antibody activity. Importantly, ZsG is expressed at moderate cellular levels, supporting its utility as a non-dominant intracellular target.

The present study introduces a conceptual and technical advance that establishes a framework for targeting intracellular tumor antigens. Beyond the specific antigen examined, the key contribution lies in the development of a platform enabling unbiased identification of antibodies against native intracellular epitopes, with broad applicability to endogenous tumor antigens. We believe that our work contributes to establishing the intracellular tumor proteome as a source of actionable targets.

Among the tumor-reactive antibodies isolated by our pipeline was a monoclonal mouse IgG1 antibody targeting ZsG. We confirmed ZsG as the cognate antigen of this anti-ZsG mAb using multiple orthogonal assays, including immunoprecipitation, immunofluorescence microscopy and flow cytometry.

Immunoprecipitation of 4T1-ZsG soluble cell extracts with the mAb produced a distinct band on SDS-PAGE that was absent from control 4T1 extracts. This band was excised and identified by mass spectrometry as ZsG protein with very high abundance. Although co-localization staining with a commercial anti-ZsG antibody was not performed, the combination of these independent validation methods provides strong confidence in the antigen specificity of the isolated mAb.

The anti-ZsG mAbs appear to target a structural epitope on the ZsG epitope. Unlike immunoprecipitation, which is traditionally performed under native conditions, the anti-ZsG mAbs did not react with the ZsG band in Western blots, where the S_105_-4T1-ZsG (a 105,000×g high speed spin supernatant of 4T1-ZsG homogenate, which contains the intracellular, soluble antigen pool) was resolved on a denaturing SDS-PAGE. Neither did the anti-ZsG antibody react in ELISA, where the target soluble extract of 4T1-ZsG was used to coat plates. Consequently, we show that antibodies targeting structural epitopes may require native methodologies for effective detection and might be overlooked when using standard protocols. Isolating antibodies targeting structural epitopes may also be important for the generation of mAbs with potential clinical significance, such as enzyme-neutralizing mAbs.

We further demonstrate the strict intracellular compartmentalization of the ZsG protein. Antibodies against ZsG isolated from our pipeline failed to stain live 4T1-ZsG cells but readily labeled the antigen after cell fixation and permeabilization. Furthermore, no secretion of ZsG was detected in the supernatant of actively dividing 4T1-ZsG cultures. These findings indicate that anti-ZsG immune responses target a genuinely intracellular protein and thereby contribute to a broader and more inclusive understanding of antigenic determinants in cancer.

To model the case of yet unknown anti-tumor antigens, we have used a soluble protein extract prepared from the mouse 4T1-ZsG tumorigenic cell line and labeled it *in vitro* with biotin. This labeled extract was used to tag anti-ZsG hybridoma, where we show coincidence of fluorescence and binding of biotinylated extract. A similar approach using a biotinylated tumor cell line extract, or direct tumor extract, can be used to probe hybridomas derived from TDLNs. The premise is, that the *in vivo* tumor proteome mirrors that of the tumorigenic mouse 4T1 cell line used for engraftment and tumor elicitation. Similar to the anti-ZsG antibodies, newly isolated antibodies from the screen for anti-tumor antibodies, could be used in immunoprecipitations from 4T1 soluble extracts, coupled to mass spectrometry identification. Since Protein L binds the kappa light chain and does not interfere with the antigen-binding site, we found it useful for our immunoprecipitation experiments. In particular, Protein L allows a uniform capture strategy for hybridomas of different isotypes. We note however that Protein L does not bind all light chain families with equal efficiency.

Notably, an anti-ZsG antibody consistently altered tumor growth trajectories relative to controls, where progressive tumor growth was seen, suggesting that intracellular antigen targeting can, under appropriate conditions, engage functional anti-tumor mechanisms *in vivo*. Treatment was initiated in early established and palpable tumors, providing a defined context for evaluating antibody activity. Importantly, the aim of the study was to evaluate antibodies isolated through the endogenous B cell-derived hybridoma platform described herein. Future studies comparing independently derived or commercial anti-ZsG antibodies, as well as evaluating endogenous intracellular tumor antigens, will help determine the generalizability and mechanistic basis of the observed effects. These findings establish a foundation for such further studies assessing efficacy across larger cohorts and controlled tumor burdens.

In order to confirm the potential of translating our suggested pipeline to human B cells, we have used the same NS0 myeloma fusion partner, to generate heterologous human/mouse hybridomas with lymphocytes derived from human (children) tonsils due to pathologies. Separately, human peripheral blood mononuclear cells (PBMCs) were used as well. Despite their hybrid chromosomal composition, fusion events between possibly activated human B cells and murine NS0 have resulted in the emergence of hybridomas, where co-expression of BCR and secretory antibodies was again observed, as in the murine model system. Tonsil-derived monoclonal hybridomas proved more stable than PBMCs, with viability sustained for many weeks. The improved viability and stability of tonsil-derived hybridomas, may be linked to the “activated” state of tonsil B cells, as these tonsils arise from pathologies manifesting enlarged nodes. Meanwhile, hybridomas derived from human PBMCs were sourced from healthy non-challenged donors, and these hybridomas likely represent predominantly quiescent non-activated lymphocyte populations. Of note, the majority of tonsillar hybridomas were of IgM isotype, consistent with the source IgM B lymphocytes isotype, whereas in the mouse 4T1 TNBC tumor model system, we have shown that TDLN-derived B cells display activated germinal center markers with a distinct bias toward IgG1 BCR expression. The activation state of sourced lymphocytes in hybridizations, may also support the persistent dual expression of BCR and secreted antibodies, in newly generated hybridomas. Much like in our mouse model, this phenotypic duality in the heterologous human/mouse hybridomas may facilitate the pipeline described herein, enabling the enrichment of human/mouse hybridomas that bind specific tumor antigens residing in soluble tumor extract.

In the mouse system, we observed a striking disparity between traditional ELISA screening and our BCR-based functional binding pipeline. Conventional ELISA screening of mouse hybridomas failed to detect anti-ZsG antibodies, whereas clear binding signals were readily observed when we applied our functional assay, which assesses direct recognition of native ZsG by the hybridoma B cell receptor (BCR). This discrepancy highlights a key limitation of traditional hybridoma screening: ELISA-based methods frequently miss antibodies that recognize natural, structural (conformational) epitopes. Such antibodies likely represent an appreciable fraction of the total anti-tumor response. We attribute this bias to partial denaturation of antigens during the ELISA plate-coating process, an effect that occurs regardless of the plate surface used. We therefore propose that the generation of heterologous human/mouse hybridomas capable of dual BCR and secretory antibody expression may better facilitate the identification of clinically relevant human anti-tumor B cell responses, including those directed against native epitopes.

The mechanisms by which antibodies targeting intracellular antigens exert anti-tumor effects remain incompletely understood. In this study, we focused on the functional outcomes of antibody activity *in vivo* rather than on delineating precise mechanisms of action. Several non-mutually exclusive mechanisms may nevertheless explain the observed therapeutic effects, with ZsG serving as a model intracellular tumor antigen. First, intracellular antigens released from dying tumor cells can form immune complexes with circulating antibodies, thereby facilitating Fc receptor-mediated uptake by antigen-presenting cells and enhancing cross-presentation to CD8^+^ T cells (2, 22). Second, tumor-reactive B cells and/or antibody-antigen complexes may promote CD4^+^ T cell priming via MHC class II pathways (3, 60). Third, aberrant trafficking or secretion of intracellular proteins may transiently expose epitopes on the cell surface, rendering them accessible to antibody-mediated effector functions (61–63).

The observation that control IgG1 antibodies accelerated tumor growth suggests that Fc-dependent modulation of the tumor microenvironment may also play a role, although this requires further investigation. Future studies will be necessary to delineate the relative contribution of these mechanisms and to establish the dependence on Fc effector functions.

The proposed functional workflow described herein may be used to further elucidate tumor-immune system interactions and identify new tumor antigens, as reflected by the host B cell response. Collectively, our findings establish a framework for systematically accessing the intracellular tumor antigen repertoire through endogenous B cell responses, providing a foundation for future mechanistic and therapeutic studies.

## Materials and methods

### Mice, *in vivo* tumor models and lymphocyte isolation from lymph nodes

4T1 cells, modeling TNBC, were sourced from ATCC and cultured as recommended by the supplier. Female BALB/c mice were purchased from Harlan Laboratories, Israel. Mice were bred and maintained under SPF-SOPF conditions, in compliance with all ethical regulations under supervision of the Tel Aviv University Committee for the Use and Treatment of Laboratory Animals.

For tumor induction and lymphocyte preparation from lymph nodes, cultured 4T1 or 4T1-ZsG cells (10^5^-10^6^) were injected into the left mammary fat pad (#4) of 8-11-week-old mice in 100-150 µl PBS. After 10–23 days, mice were sacrificed. Nodes from the inguinal TDLN, contralateral, or distal lymph nodes were excised into DMEM (Sartorius 01-052-1A) or PBS/0.1% BSA. Lymphocytes were extracted by mechanical dissociation followed by passage through a 40-µm cell strainer. Induced tumors were monitored using caliper measurements. Tumor volume was calculated (mm³) using the formula 0.5 × length × width². Mouse weights were recorded twice weekly. The two therapeutic monoclonal antibodies used for mouse *in vivo* experiments comprised the isotype with kappa light chains. One was specific for ZsG and another control antibody recognizing an unrelated intracellular antigen that is not specific to tumor cells. Large-scale purification of these two therapeutic antibodies used for treating mouse tumors, was performed in bioreactors (Sarstedt, miniPERM™) and purified over a Protein A cartridge (Cytiva, HiTrap Fibro PrismA, 17-5498-56) at the Weizmann Institute of Science, Life Sciences Core Facilities-Antibody Engineering Unit. Aliquots were stored at -20 °C with no added cryoprotectant. Reactivity of the produced antibodies was confirmed by immunofluorescence assays using fixed and permeabilized 4T1-ZsG versus 4T1 (control) cells. Therapeutic antibody injections were delivered intravenously via the tail vein at 100 µg/mouse in 130-200 µl PBS, twice weekly. At endpoint, tumors were excised and weighed. Tumors arising from 4T1-ZsG cells were confirmed to sustain expression of the ZsG protein by subjecting the tumors to Collagenase IV (Thermo Fisher 17104019) and DNase I (Promega M6101) treatment, followed by passing through a 40 µm cell strainer and flow cytometry assessment of ZsG fluorescence in the dissociated tumor cells.

### Lentiviral transduction of ZsG into 4T1 cells

The bicistronic pLVX-EF1a-IRES-ZsG vector (Clontech/Takara Bio USA, 631982) was used for ectopic expression. Packaging was performed using second-generation helper plasmids psPAX2 and pMD2.G (gift of Didier Trono). Following lentiviral transduction, 4T1 cells were enriched to 100% ZsG-positive by FACS, to select for stable ZsG expressors and avoid tumor escape in the orthotopic model treated with anti-ZsG antibodies.

### ZsG relative cellular abundance in 4T1-ZsG cell line, by mass spectrometry

Label-free quantification of ZsG protein in 4T1 versus 4T1-ZsG cells was determined in triplicates by two-dimensional (2D) MS/MS (Smoler Proteomics Unit, Technion, Israel Institute of Technology). Cells were detached from plates by a 10 min incubation with PBS/2 mM EDTA, followed by plate agitation and scraping. Approximately 300,000 cells, in triplicates, were trypsinized, separated on a reversed-phase C18 HPLC column and analyzed by 2D MS/MS using the Orbitrap Exploris™ 480 Mass Spectrometer (ThermoFisher Scientific). Protein identification was based on a minimum of two peptides per protein. MaxQuant and Perseus 1.6.7.0 software (Mathias Mann’s lab, Max Planck Institute) were used to obtain normalized intensities and perform statistical analyses.

### Preparation of intracellular, soluble protein extract from 4T1 and 4T1-ZsG cell cultures (S_105_-4T1 and S_105_-4T1-ZsG)

140 mm tissue culture plates of confluent 4T1 or 4T1-ZsG cells were washed four times with PBS, with the final wash including 2 mM EDTA. No protease was used for cell detachment. Cells were scraped in 5 ml/plate of 0.5× PBS/2 mM EDTA containing complete Protease Inhibitor (Sigma-Aldrich/Roche 11836153001). Following low-speed centrifugation, cells were resuspended in 1-1.5 ml/plate equivalent of the same buffer. Cell suspension aliquots were homogenized to completion by sonication (three bursts with 30-second intervals on ice). Homogenates were then subjected to differential centrifugation at 2,000×g for 10 minutes followed by separation of the supernatant and by subjecting it to another round of centrifugation at 105,000×g for 1 hour at 16 °C. The supernatant of the final high-speed spin, representing the soluble intracellular protein/antigen pool was separated, filtered through 0.45-µm filter and stored in aliquots at -20 °C with no added cryoprotectant. Protein concentration (1–5 mg/ml) was determined using Bradford Protein Dye Reagent (Bio-Rad, 5000002), with BSA as standard.

### Flow cytometry analysis of B cells reacting against the ZsG antigen in TDLNs of 4T1-ZsG tumors

Mice bearing 4T1-ZsG tumor cells and 4T1 control tumor cells were sacrificed 14–18 days after tumor engraftment. TDLNs were extracted in 2 ml/node of PBS/0.1% BSA, mechanically dissociated, passed through a 40 µm cell strainer and washed twice by centrifugation. The lymphocyte population was identified by flow cytometry based on forward scatter (FSC) and side scatter (SSC) characteristics (**Supplementary Figures 1B, C**).

Staining for ZsG was performed by incubation of lymphocytes extracted from tumors with 75 µg of S_105_-4T1 or S_105_-4T1-ZsG extract (representing the intracellular, soluble protein/antigen pool of 4T1 cultured cells or 4T1-ZsG cultured cells, prepared by a 105,000×g high speed spin supernatant of the corresponding cell homogenate). After 30 minutes, cells were washed twice with PBS/0.1% BSA and stained with anti-mouse CD19-VB (REAfinity™ antibody (Miltenyi, 130-111-889). Acquisition and analysis were carried out using an Attune NxT flow cytometer (ThermoFisher Scientific, A24859).

### Fusion of murine NS0 myeloma cells with lymphocytes extracted from lymph nodes of 4T1 and 4T1-ZsG tumor bearing mice

Hybridization was performed essentially as described by Kohler and Milstein (35). Non-secreting NS0 murine myeloma cells were provided by C. Milstein and N. Smorodinsky. Cells were cultured in DMEM (Sartorius 03-020-1A) supplemented with 15% horse serum (Sigma-Aldrich, H1270), 2 mM glutamine, 1% OPI (Sigma-Aldrich Hybrimax, O5003), and 0.4% Penicillin/Streptomycin/Nystatin (Biological Industries 03-032-1B).

For fusion, TDLN lymphocytes were combined with NS0 myeloma cells at a ratio of 4:1. PEG 1450 (Sigma Aldrich Hybrimax P7181) was used for fusion and 2% HAT (Sartorius 03-080-1B) for selection. Cultures were weaned from HAT at day 10-14. For single-cell cloning, murine IL-6 at 100 U/ml was added.

### Heterologous fusion of murine NS0 myeloma cells with human tonsil and blood lymphocytes

Human peripheral blood mononuclear cells (PBMCs) were isolated from anonymized leukocyte-enriched blood units obtained from healthy volunteer blood donors through Magen David Adom (MDA) National Blood Services, Israel. The blood units were provided to the investigators in a fully de-identified form and used in accordance with institutional policies and applicable regulations governing research involving anonymized human biological specimens. Human tonsil lymphocytes were obtained from anonymized tonsil specimens collected from patients undergoing tonsillectomy under a protocol approved by the Institutional Review Board of Tel Aviv University and conducted in accordance with the Declaration of Helsinki. Written informed consent was obtained from all participants or, in the case of minors, from their legal guardians. The tonsil specimens were provided to the investigators in a fully de-identified form. Tonsils were mechanically dissociated in DMEM (Sartorius 01-052-1A) and passed through a 40 µm cell strainer. Tonsil lymphocytes and PBMCs were further enriched on Ficoll (MP Biomedicals, LSM™ MP0850494X). The enriched lymphocyte fractions were washed in DMEM and fused with NS0 myeloma cells at a ratio of 4:1. Subsequent plating and cloning procedures were performed as described for murine hybridomas.

### Antibody collection from monoclonal hybridomas

Cells were washed three times with DMEM and resuspended in Serum Free Medium (ThermoFisher Scientific, Hybridoma-SFM 12045084) supplemented with Penicillin/Streptomycin/Nystatin. After 2–3 days, cells were pelleted by centrifugation and the supernatant was collected. HEPES (1 M, pH 7.4) was added to a final concentration of 25 mM, and the medium was stored at 4 °C.

### Labeling of 4T1 and 4T1-ZsG soluble protein extracts, by Maleimide conjugation to Biotin

Maleimide-PEG2-Biotin (ThermoFisher Scientific TS-A39261) was dissolved in water-free DMSO or PBS. The protein-to-crosslinker ratio followed manufacturer recommendations. Reactions were performed at pH 6.5 to favor sulfhydryl conjugation. Excess reagent was quenched with 50 mM DTT, and samples were clarified by centrifugation (20,000×g, 3 minutes). Conjugated extracts were purified using PD-10 gel filtration columns (Sigma Aldrich GE17-0851-01) equilibrated with PBS/2 mM EDTA.

### Flow cytometry analysis of membranal BCR expression on hybridomas

Bulk populations of hybridized cells before single-cell cloning, or monoclonal populations, were washed two times with PBS by centrifugation. Final pellet was resuspended in 50 µl of PBS/0.1% BSA/0.1% Sodium Azide (CSB-cell staining buffer) and stained with anti-mouse-IgM-PE-REA REAfinity (Miltenyi, 130-116-209) for IgM hybridoma, or anti-mouse-IgG1-PE REAfinity (Miltenyi, 130-117-057) for IgG1 hybridoma. After 10 min of incubation, 300 µl of CSB were added and the stained cells were analyzed using flow cytometry (Attune NxT, ThermoFisher Scientific, A24859). Negative control for the monoclonal IgM hybridoma stained with anti-IgM-PE was a monoclonal IgG1 hybridoma population stained with the same anti-IgM-PE antibody. Similarly, negative control for the IgG1 hybridoma stained with anti-IgG1-PE was a monoclonal IgM hybridoma population stained with the same anti-IgG1-PE antibody.

### RT-PCR and Sanger sequencing

To sequence hybridoma DNA encoding antibodies, RNA was extracted (Zymo Research ZR-R1050). Oligo dT primers, (dT)20VN, anchoring to the 5’ end of the poly-A tail, were added to the RNA. The mixture was heated at 70 °C for 5 minutes and annealed on ice for 5 minutes. RNase H minus Reverse Transcriptase (RevertAid, ThermoFisher Scientific, EP0452) was added to initiate reverse transcription, followed by incubation at 42 °C for 1 hour. The resulting cDNA was amplified by polymerase chain reaction (PCR) using high-fidelity Hot Start PrimeSTAR polymerase master mix (Takara Bio R045A), with isotype-specific reverse primers and degenerate forward primers (64). PCR cycling conditions were: (1) pre-denaturation at 98 °C for 30 sec; (2) denaturation at 98 °C for 10 sec; (3) annealing at 58 °C for 5 sec; (4) extension at 72 °C for 5 sec; steps 2–4 were repeated for 35 cycles; followed by (5) a final extension at 72 °C for 1 min. Following agarose gel electrophoresis, amplified heavy and light chain bands were excised and purified (ZymoResearch Gel DNA Recovery Kit D4008). Samples were submitted for Sanger sequencing on an ABI 3500xl Genetic Analyzer at the Tel Aviv Life Sciences Core Facility.

### MACS^®^ column enrichment

MACS buffer consisted of PBS/0.5% BSA/2 mM EDTA. Cells were labeled with PE-conjugated antibodies and anti-PE nanobeads (Miltenyi), followed by LS column separation as indicated by the manufacturer. Antigen enrichment was performed using biotinylated extracts and Streptavidin-PE prior to magnetic selection.

### FACS-aided single cell cloning of anti-ZsG and anti-tumor-antigen hybridomas

Hybridomas were stained with antigen extracts and sorted using a BD FACSAria III. Highly ZsG fluorescing single cells were deposited into individual wells for clonal expansion.

### Fluorescence microscopy of 4T1-ZsG cells stained with anti-ZsG antibodies

4T1 or 4T1-ZsG cells were washed and either fixed with 150 µl of methanol-free 4% paraformaldehyde (PFA) for 25 minutes and then permeabilized with 150 µl of PBS/NP-40 0.2% for 20 minutes, or left unfixed and incubated with PBS alone. Protein A affinity-purified anti-ZsG monoclonal antibody, at 10 µg/ml in PBS containing 1% BSA, was used to stain both the fixed/permeabilized and the non-fixed wells. Following a one-hour incubation, wells were washed three times with PBS and stained with anti-mouse-IgG1-PE REAfinity™ (Miltenyi 130-117-057). Following a 15-minute incubation, cells were washed three times with PBS, and imaged using a digital fluorescence microscope (Bio-Rad ZOE Fluorescent Cell Imager, 1450031) in the ZsG, PE and bright field channels. Merged images of the ZsG and PE fluorescence channels were generated using ImageJ (NIH).

### Direct bulk immunoprecipitation of ZsG

Protein L magnetic beads (ThermoFisher Scientific, Pierce™ Protein L Magnetic Beads TS-88849) were incubated with antibodies and S_105_ extracts (representing the intracellular, soluble protein/antigen pool of 4T1 cultured cells or 4T1-ZsG cultured cells, prepared by a 105,000×g high speed spin supernatant of the corresponding cell homogenate). Following incubation of antibody-loaded beads with S_105_ extracts, the Protein L beads were washed 3X by centrifugation and visualized under a fluorescent microscope. The commercial mouse IgG1 isotype control (Biolegend, BLG-401402), which does not bind mouse antigens, was used as negative control. The anti-ZsG antibody isolated through our pipeline was Sanger-sequenced prior to the IP experiment. Both our anti-ZsG and the commercial isotype control antibody used in this set of experiments harbor kappa light chains.

### Direct immunoprecipitation of ZsG and resolution of products

200 µl of protein L magnetic beads (ThermoFisher Scientific, Pierce™ Protein L Magnetic Beads TS-88849) were washed with PBS on magnet and incubated for 5 min in 1 ml blocking solution (PBS/0.1% BSA/2mM EDTA). Beads were incubated on orbital shaker for 2 hours at RT with 8 ml Serum Free Medium collected from an anti-ZsG monoclonal hybridoma, supplemented with 0.1% BSA. After 3X washings with blocking solution on magnet, beads were equally distributed into 3 tubes. One tube was resuspended in PBS/2My mM EDTA. S_105_ extracts prepared from either 4T1 or 4T1-ZsG cell homogenates (representing the intracellular, soluble protein/antigen pool of either 4T1 cultured cells or 4T1-ZsG cultured cells and resulting from a 105,000×g high speed spin supernatant of the corresponding cell homogenate), were thawed, complemented with 2 mM EDTA, and clarified by centrifugation. Approximately 1.5 mg of each extract was added to the remaining 2 bead-containing tubes. Incubation at RT proceeded for 45 minutes with orbital shaking. Beads were washed 3X with PBS and the pellets were resuspended in sample buffer containing 2% SDS, 4M Urea and 0.5M DTT. Following heating, material was loaded onto a Novex precast 4-20% Tris-Glycine wedge gel (ThermoFisher Scientific, XP04202BOX), electrophoresed and stained with colloidal CBB-R250 (Tivan, Fast Seeband, SB050). Bands were excised at the migration chromatographic Retention factor (Rf) corresponding to differentially migrating bands between immunoprecipitation performed with 4T1 versus 4T1-ZsG extracts.

### Identification of immunoprecipitated products by mass spectrometry

Excised samples from Coomassie-stained protein SDS-PAGE gels were sent to the Smoler Proteomics Unit, Technion-Israel Institute of Technology. Samples were digested by trypsin, analyzed by LC-MS/MS on Q Exactive™ Plus Hybrid Quadrupole-Orbitrap™ Mass Spectrometer (ThermoFisher Scientific) and identified by Discoverer 2.4 software against Mouse Uniprot Protein Database and a decoy database, to determine false discovery rates. Protein matched identification was carried out with SEQUEST^®^ (ThermoFisher Scientific). The ZsG sequence was added manually to the expected repertoire.

### Screening for human antibody secretion from heterologous hybridomas, by ELISA

ELISA 96-well plates (Greiner, high bind 655061) were coated overnight at 4 °C with 50 µl serum-free hybridoma supernatants. Goat Anti-Human IgM, Fc5μ fragment specific, conjugated to Horseradish Peroxidase (Jackson ImmunoResearch Europe Ltd., AffiniPure™ 109-035-043) was then used. Plates were developed with Tetramethylbenzidine (Biotest, TMB TM4500) and stopped with H_2_SO_4._ Results were acquired on a plate reader (BioTek/Agilent, SynergyTM H1 BID S1LFA). A well coated with serum-free medium was used as blank.

### SDS-PAGE to confirm human antibody secretion

Collected monoclonal antibodies were concentrated by ultrafiltration on 10kDa MWCO filter unit (Amicon, UFC901024) and then 100kDa MWCO (Amicon, UFC910024). Approximately 100 µg of retained protein were denatured and reduced before loading onto a Novex precast 4-20% Tris-Glycine SDS-PAGE wedge gel (ThermoFisher Scientific, XP04202BOXG). Gels were stained with colloidal CBB-R250 (Tivan, Fast Seeband SB050).

### Data visualization and analysis

Statistical analyses were performed using GraphPad Prism 10. Flow cytometry data were analyzed using FlowJo v10. Figures were generated using BioRender.

## Data Availability

The original contributions presented in the study are included in the article/Supplementary Material, further inquiries can be directed to the corresponding author/s.
