# Supplementary material for: B cell receptor–based hybridoma enrichment enables discovery of antibodies against intracellular tumor antigens
Source: Front Immunol. 2026 Jul 30;17:1867422. doi: 10.3389/fimmu.2026.1867422 (PMC13469021; doi:10.3389/fimmu.2026.1867422)
Supplement: Supplementary file 1 [file DataSheet1.pdf]

B CELL RECEPTOR (BCR)-BASED HYBRIDOMA ENRICHMENT ENABLES DISCOVERY OF ANTIBODIES AGAINST INTRACELLULAR TUMOR ANTIGENS

Supplementary Information

Supplementary Fig. S1: Characterization of lymphocyte populations in contralateral lymph nodes of 4T1 tumor-bearing mice and gating strategies.

Supplementary Fig. S2: Stable expression of ZsGreen1 in 4T1 murine breast cancer cells following lentiviral transduction

Supplementary Fig. S3: Enrichment of germinal center B cells from tumor-draining lymph nodes of 4T1-ZsG tumor-bearing mice

Supplementary Fig. S4: Somatic mutation analysis of anti-ZsGreen1 monoclonal antibody heavy-chain sequences relative to germline

Supplementary Fig. S5: Immunoglobulin isotype expression in human-mouse hybridomas derived from PBMC-NS0 cell fusion

Supplementary Table S1: Characterization of tumor antigen-reactive hybridomas following MACS enrichment and single-cell cloning

Supplementary Figure S1

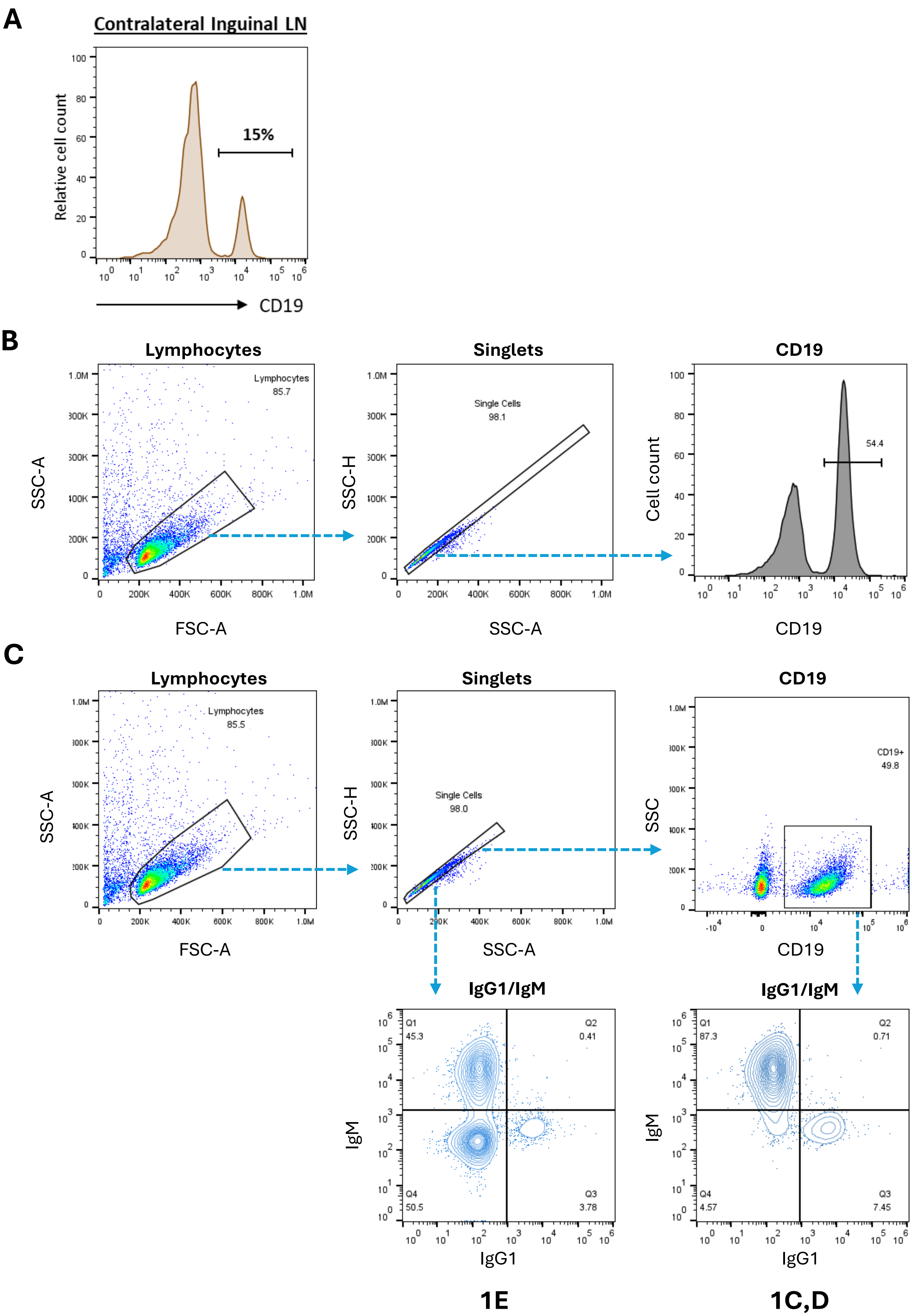

Supplementary Figure S1: Characterization of lymphocyte populations in contralateral lymph nodes of 4T1 tumor-bearing mice and gating strategies. **A.** Representative flow cytometry analysis of CD19^{+} B cell frequency in the contralateral inguinal lymph node (LN; non-tumor-draining) of a mouse orthotopically engrafted with 4T1 tumor cells, assessed 13 days post engraftment. **B.** Gating strategy for Fig. 1A,B. **C.** Gating strategy for Fig. 1C-E.

Supplementary Figure S2

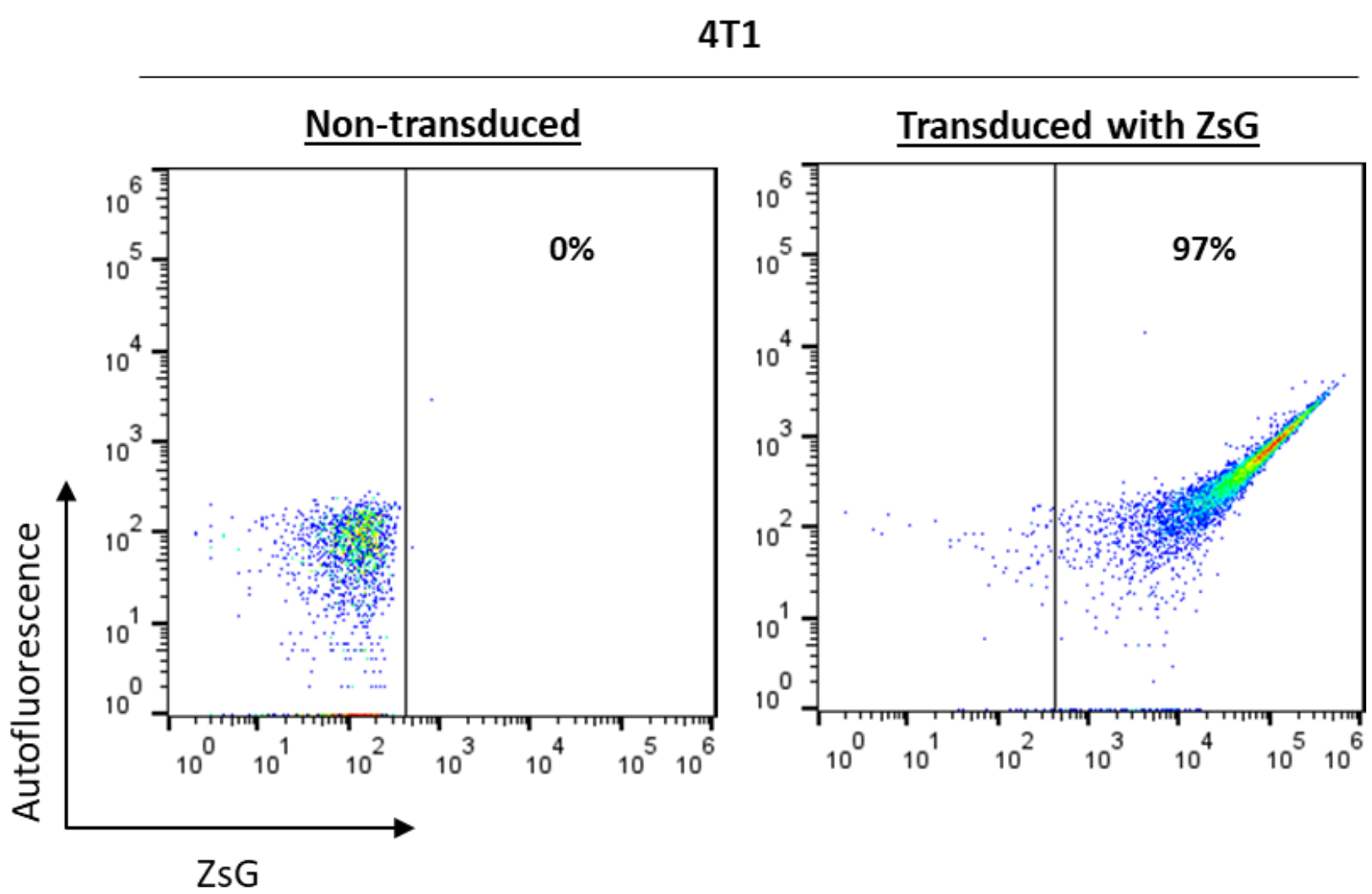

Supplementary Figure S2: Stable expression of ZsGreen1 in 4T1 murine breast cancer cells following lentiviral transduction. Representative flow cytometry analysis of ZsGreen1 (ZsG) expression in 4T1 cells following lentiviral transduction (right) compared to non-transduced parental 4T1 cells (left).

Supplementary Figure S3

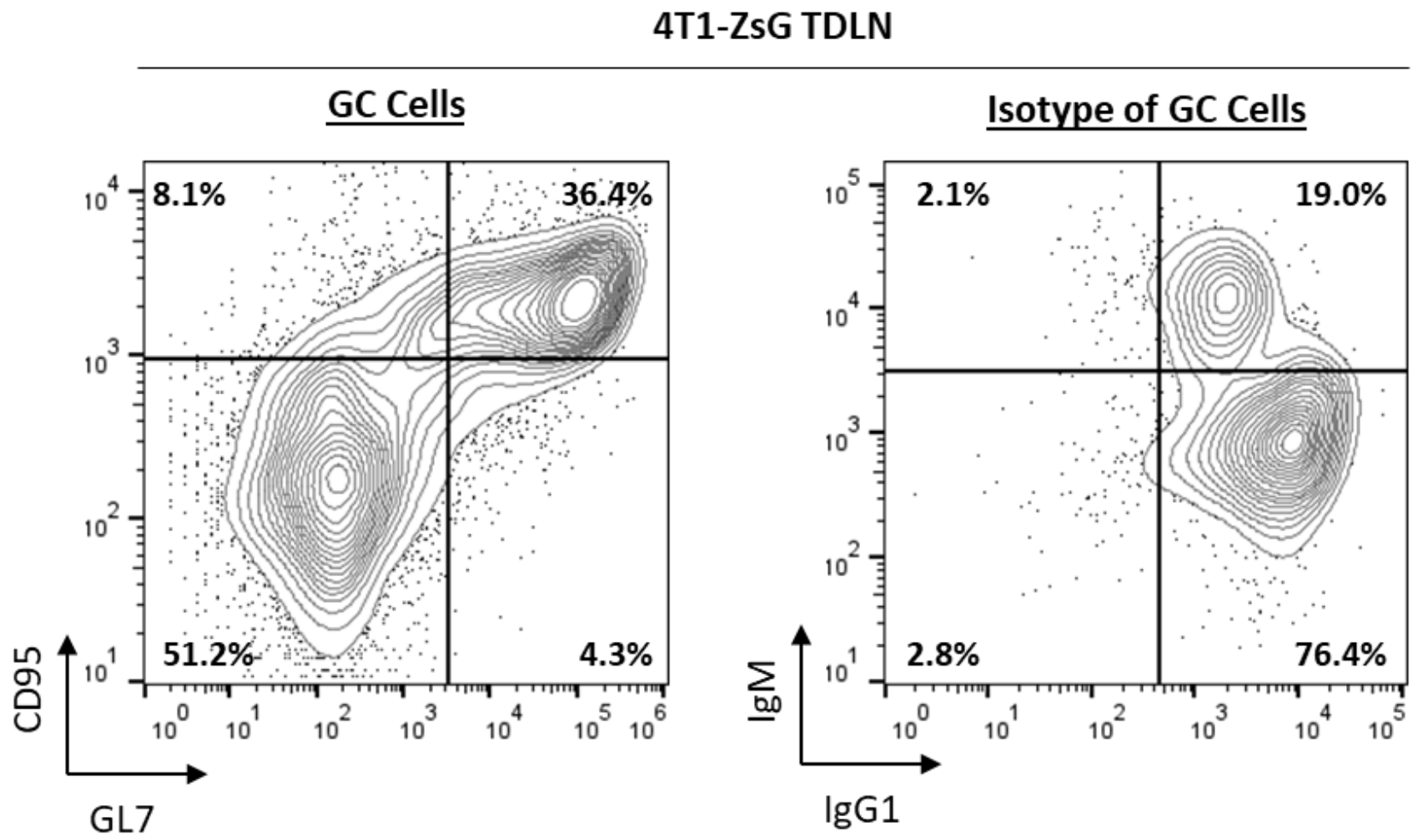

Supplementary Figure S3: Enrichment of germinal center B cells from tumor-draining lymph nodes of 4T1-ZsG tumor-bearing mice. Representative flow cytometry analysis of lymphocytes isolated from the tumor-draining lymph node (TDLN) of a mouse bearing 4T1-ZsG tumors, 12 days post implantation, following enrichment for germinal center (GC) B cells using PNA bead selection.

Supplementary Figure S4

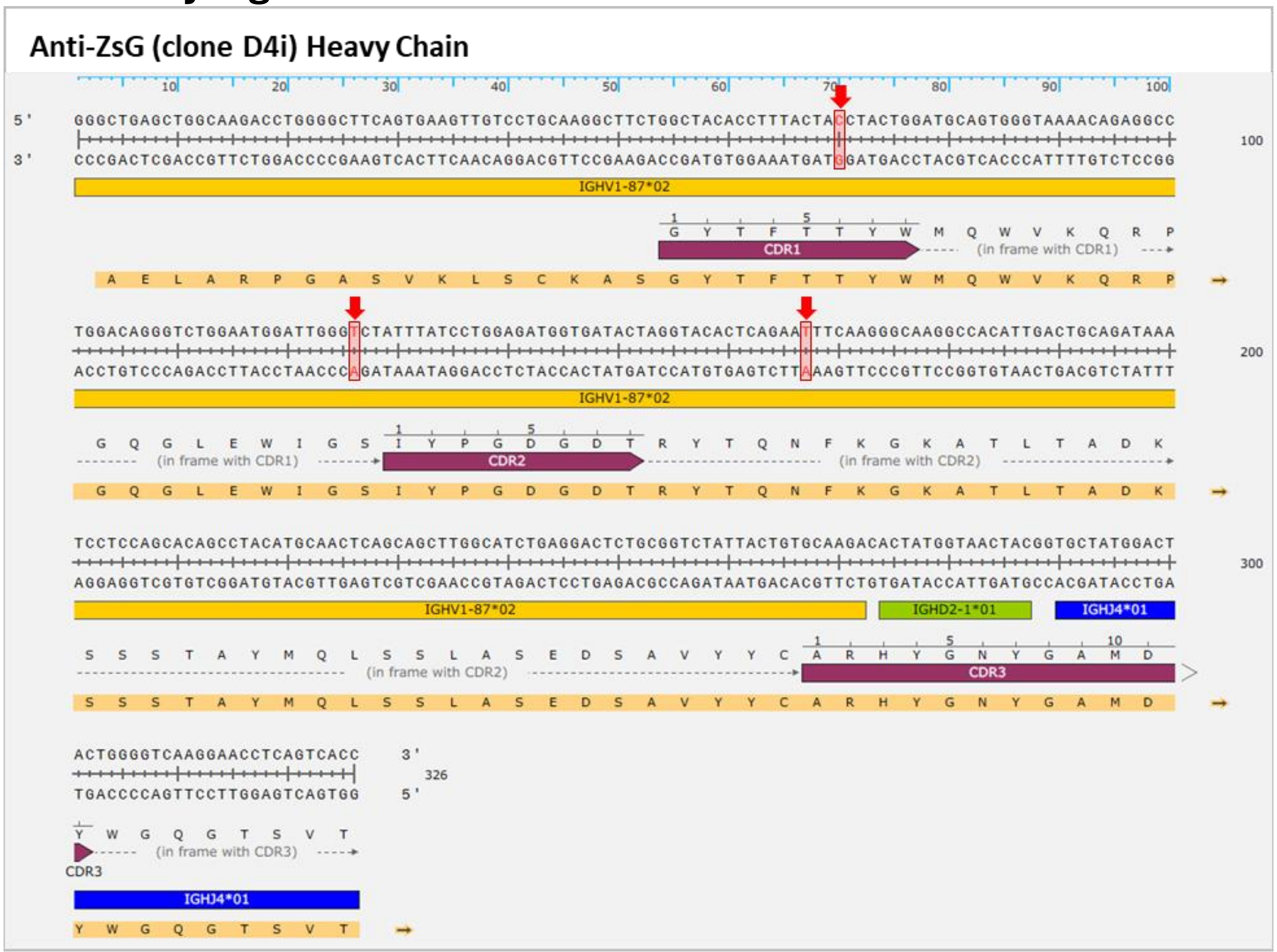

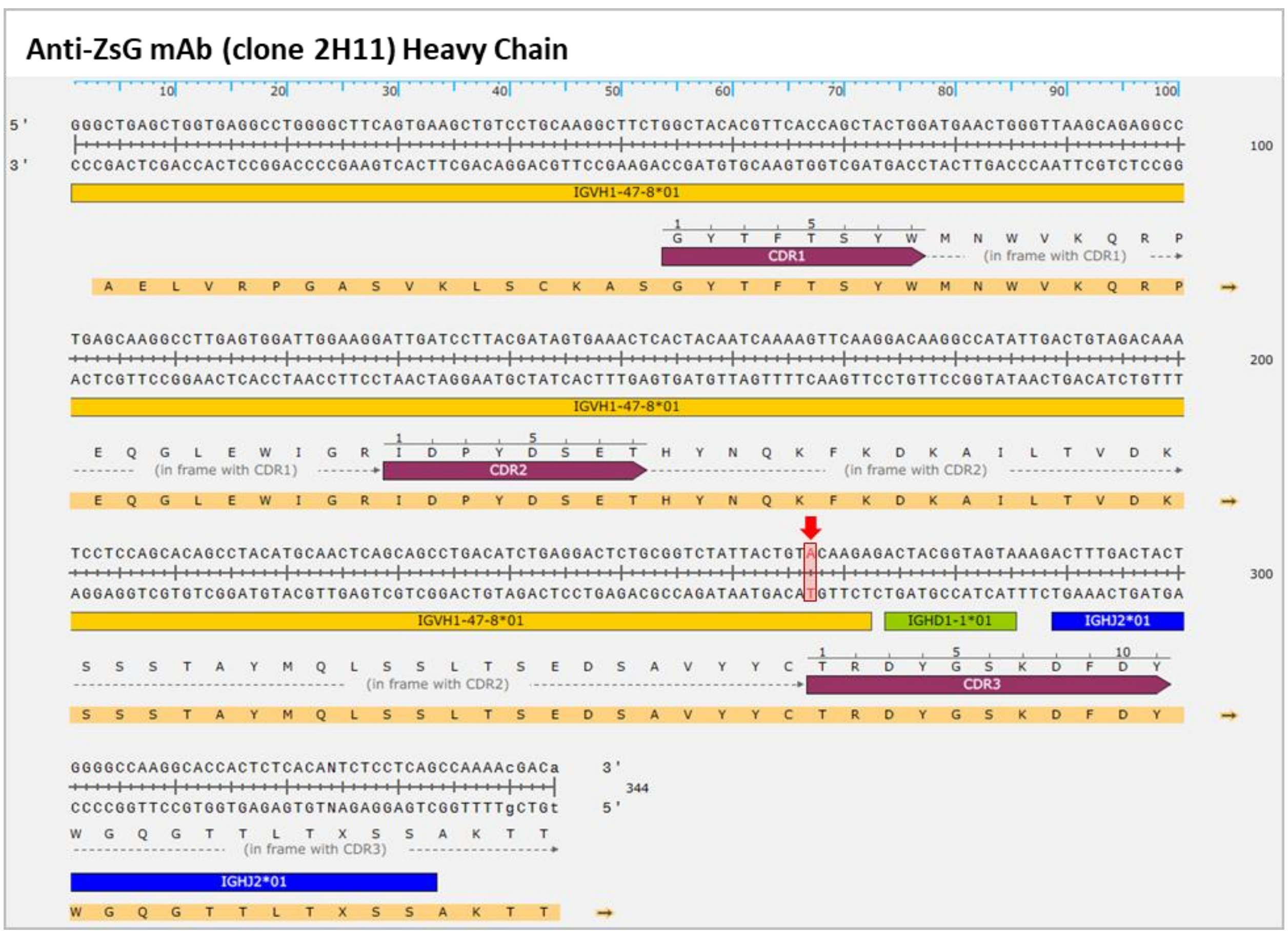

Supplementary Figure S4: **Somatic mutation analysis of anti-ZsGreen1 monoclonal antibody heavy-chain**

sequences relative to germline. Alignment of immunoglobulin heavy-chain variable region sequences from two anti-ZsGreen1 monoclonal antibodies (mAbs) with their respective germline genes. Somatic mutations are highlighted in red boxes.

Supplementary Figure S5

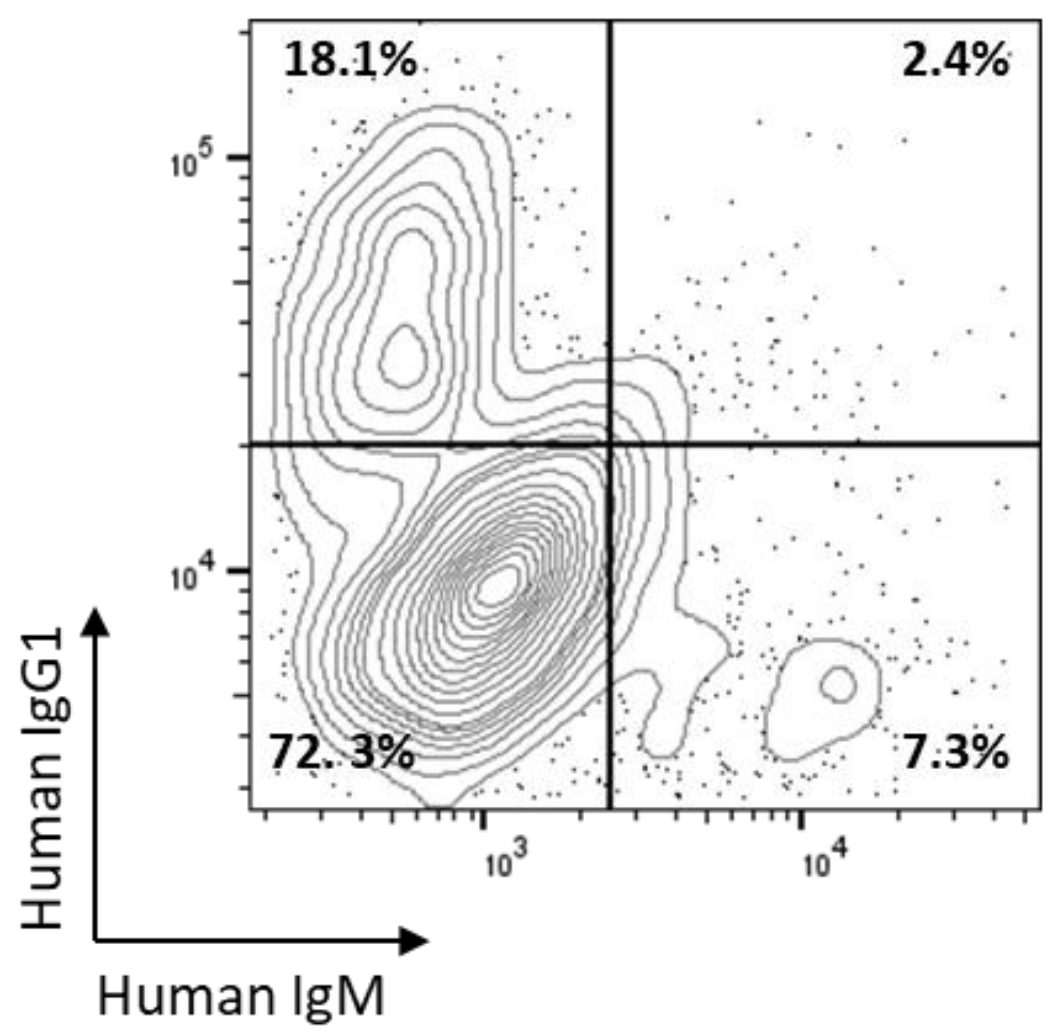

Supplementary Figure S5: Immunoglobulin isotype expression in human-mouse hybridomas derived from PBMC-NS0 fusion. Representative flow cytometry analysis of IgM and IgG expression in hybridomas generated by fusion of human PBMCs from healthy donors with NS0 mouse myeloma cells, assessed 9 days post hybridization.

Supplementary Table S1

Mouse Isotype	Number of clones	Number of clones that stain fixed 4T1	Percentage of clones that stain fixed 4T1
**IgG1**	15	4	27
**Ig2a**	2	2	100
**IgG2b**	1	0	0
**IgM**	15	2	13
**Other**	13	0	0

Supplementary Table S1: Characterization of tumor antigen-reactive hybridomas following MACS enrichment and single-cell cloning. Summary of monoclonal hybridomas enriched for tumor antigen binding by magnetic-activated cell sorting (MACS® column), followed by single-cell cloning. Antibody isotype and staining performance on fixed and permeabilized 4T1 cells were evaluated by immunofluorescence. “Other” denotes isotypes distinct from IgG1, IgG2a, IgG2b, or IgM.
